# Supplementary material for: Structural validity and reliability of the patient experience measure: A new approach to assessing psychosocial experience of upper limb prosthesis users
Source: PLoS One. 2021 Dec 28;16(12):e0261865. doi: 10.1371/journal.pone.0261865 (PMC8714100; doi:10.1371/journal.pone.0261865)
Supplement: S1 Appendix — (DOCX) [file pone.0261865.s001.docx]

**S1 Appendix**

**Detailed description of item revision process**

Prior to cognitive testing with subjects in the current study, who were not using sensory restoration systems, we modified the introductory instructions, removing reference to sensation condition which had been part of the original PEM for the prior research study. The instructions were changed to read, “When you answer the following questions, please consider your experience over the last week. If you did not perform the activity, make your best guess as to how you would have felt about performing the activity with your prosthesis.”

For easier telephone administration, we modified the response scales to 1-5 point Likert scales, adding unique anchor words for each subscale and generating an introduction to each subscale which related to the anchor words for each subscale, respectively. We also made minor revisions to the wording of items to make them clearer and more conducive to interviewer administration.

We also added 2 new items related to self-efficacy: “Using my prosthesis to pick up fragile objects,” “Using my prosthesis to do two-handed tasks.,” We condensed several original items in the body-image subscale which reflected positive and negative aspects of the same construct; eg. “more confident and less confident”, “more whole and less whole”, and “more shy and less shy”. This resulted in the elimination of 3 items. We also generated 2 new items which we believed fit well with the construct of prosthesis efficiency; “Using a prosthesis requires concentration,” and “Using my prosthesis comes naturally.”

Cognitive interviews resulted changes to the numerical values in the response categories from 1-5 to 0-4, because it seemed to make more sense to respondents to refer to the lower boundary of the scales (anchored with words, such as, “not at all comfortable”) as “0” than for it to be assigned a value of “1”. We also changed the organization of the items, grouping all items that were positive together and separating them from the items which were negative to avoid confusion when switching between positive and negative attitudes towards the prosthesis.

Additionally, we revised the wording of several items to be more precise and uniform and to eliminate variability in responses due to differences in interpreting the method of performing specific tasks. For example, we changed “using my prosthesis to do two-handed tasks” to “Using a prosthesis to carry a laundry basket.” We modified the item, “My prosthesis is an extension of me,” to “My prosthesis is an extension of my body.” We modified the item: “Using my prosthesis comes naturally,” because it seemed to mean different things to different people, replacing it with “Using my prosthesis is not natural.” For greater clarity, we changed the word, “holding” to “grasping with” because participants explained that the act of “grasping with” better captured the action of using the prosthetic terminal device actively to open and close it to grasp. Thus, the item “Holding someone else’s hand while walking without hurting them” was revised to “Grasping someone else’s hand while walking without hurting them.” To be more specific, we modified, “Shaking hands without hurting or pinching someone” to “Opening your terminal device when shaking hands.” We found that some participants described holding hands by extending the closed prosthesis and allowing the other party to grasp hold of their prosthesis; in this way avoiding the possibility of inadvertently hurting or pinching by the terminal device.

We also added 5 new items based on feedback and conversations in the cognitive interviews. We generated new items that pertained to tasks that respondents had mentioned that they were not confident or comfortable performing with their prosthesis: “Using a knife and fork while eating out,” “Holding a dinner glass,” “Using a prosthesis to hold a child,” “Using a prosthesis to pick up a small child,” and “Tying a knot” We also added 3 items: “Relaxed,” “Vulnerable,” and “Incomplete,” related to body image and embodiment, using some of the actual words participants had used when describing how they felt with or without their prosthesis. Finally, we generated another new item, “Using a prosthesis is clumsy”, utilizing a word one of our participants had used when describing how he felt about his prosthesis use.

We also removed several items. We removed the item, “Using a prosthesis to communicate your anger through touch,” because none of the participants we interviewed endorsed this response, perhaps because the item implied violence and a positive response would not be viewed as socially acceptable. We removed the item “Using a prosthesis is slower than using my other hand,” because the term “my other hand” was not clear enough and was interpreted a variety of ways (a prosthesis or its part, a stump, a phantom limb). Additionally, it was not appropriate for bilateral amputees.

Prior to pilot testing we refined the measure based on discussion with the University of Massachusetts survey team. We changed the reference time point from “last week” to “past 4 weeks). We made minor modifications to the order and wording of the instructions to simplify them, i.e. changed “performing” to “doing” tasks and “using any number” instead of “scale”. We also made minor changes to make wording consistent across items, i.e. referring to “your prosthesis” or “my prosthesis” instead of “a prosthesis”.

After pilot testing, we made the following minor revisions based on respondent questions and interviewer feedback. We removed introductory text “For this next set of questions…” and clarified the instructions for the self-efficacy related items and one of the items. We also added the words “using a prosthesis” for all self-efficacy related items in the for consistency. Finally, we added a new response “would not/do not do”. A copy of the final measure that was field tested is shown in S2 File Appendix B.
