# Supplementary material for: Structural validity and reliability of the patient experience measure: A new approach to assessing psychosocial experience of upper limb prosthesis users
Source: PLoS One. 2021 Dec 28;16(12):e0261865. doi: 10.1371/journal.pone.0261865 (PMC8714100; doi:10.1371/journal.pone.0261865)
Supplement: S2 Appendix — Measure used in field testing. (DOCX) [file pone.0261865.s002.docx]

**S2 Appendix**

**Revised Patient Experience Measure**

**Version used in Field Testing**

**[ASK IF USER AND USED PROSTHESIS IN PAST 4 WEEKS]**

For this next question, please answer using any number from 0 to 4, where 0 is Not at All Confident and 4 is Very Confident. How confident were you in your ability to do each of the following using your prosthesis over the last 4 weeks?

|  | Not at All Confident |  |  |  | Very Confident | WOULD NOT DO/DO NOT DO [DO NOT READ] | DON’T KNOW/ NOT SURE/NOT APPLICABLE [DO NOT READ] | REFUSED [DO NOT READ] |
| --- | --- | --- | --- | --- | --- | --- | --- | --- |
| 1. Using your prosthesis to carry a small object, such as a coin, without dropping it [Please pick any number from 0 to 4 where 0 equals Not at all confident and 4 equals Very confident] | 0 | 1 | 2 | 3 | 4 | 97 | 98 | 99 |
| 1. Using your prosthesis to grasp someone else's hand while walking without hurting them | 0 | 1 | 2 | 3 | 4 | 97 | 98 | 99 |
| 1. Using your prosthesis to pick up an open plastic water bottle without dropping or crushing it | 0 | 1 | 2 | 3 | 4 | 97 | 98 | 99 |
| 1. Using your prosthesis to drink from a paper cup without dropping or crushing it | 0 | 1 | 2 | 3 | 4 | 97 | 98 | 99 |
| 1. Using your prosthesis to pick up a Ritz cracker without breaking it | 0 | 1 | 2 | 3 | 4 | 97 | 98 | 99 |
| 1. Using your prosthesis to eat with a knife and fork while in a restaurant | 0 | 1 | 2 | 3 | 4 | 97 | 98 | 99 |
| 1. Holding a dinner glass using your prosthesis | 0 | 1 | 2 | 3 | 4 | 97 | 98 | 99 |
| 1. Tying a knot using your prosthesis | 0 | 1 | 2 | 3 | 4 | 97 | 98 | 99 |
| 1. Using your prosthesis to carry a slippery object, such as a silk scarf or tie, without dropping it | 0 | 1 | 2 | 3 | 4 | 97 | 98 | 99 |
| 1. Using your prosthesis to pick up fragile objects | 0 | 1 | 2 | 3 | 4 | 97 | 98 | 99 |
| 1. Using your prosthesis to carry a laundry basket | 0 | 1 | 2 | 3 | 4 | 97 | 98 | 99 |
| 1. Trying new tasks with your prosthesis | 0 | 1 | 2 | 3 | 4 | 97 | 98 | 99 |
| 1. Opening your terminal device when shaking hands | 0 | 1 | 2 | 3 | 4 | 97 | 98 | 99 |

**[ASK IF USER AND USED PROSTHESIS IN PAST 4 WEEKS]**

Using any number from 0 to 4, where 0 is Not at All Comfortable and 4 is Very Comfortable, how comfortable were you with each of the following over the last 4 weeks?

|  | Not at All Comfortable |  |  |  | Very Comfortable | WOULD NOT DO/DO NOT DO [DO NOT READ] | DON’T KNOW/ NOT SURE/NOT APPLICABLE [DO NOT READ] | REFUSED [DO NOT READ] |
| --- | --- | --- | --- | --- | --- | --- | --- | --- |
| 1. Grasping with your prosthesis to shake hands with someone close to you [Please pick any number from 0 to 4 to indicate your comfort level, where 0 equals Not at All Comfortable and 4 equals Very Comfortable] | 0 | 1 | 2 | 3 | 4 | 97 | 98 | 99 |
| 1. Grasping with your prosthesis to shake hands with someone you just met | 0 | 1 | 2 | 3 | 4 | 97 | 98 | 99 |
| 1. Grasping with your prosthesis to shake hands with someone you know well | 0 | 1 | 2 | 3 | 4 | 97 | 98 | 99 |
| 1. Using your prosthesis to gently squeeze someone else's hand | 0 | 1 | 2 | 3 | 4 | 97 | 98 | 99 |
| 1. Using your prosthesis when embracing someone you care about | 0 | 1 | 2 | 3 | 4 | 97 | 98 | 99 |
| 1. Using your prosthesis to convey a friendly or caring touch | 0 | 1 | 2 | 3 | 4 | 97 | 98 | 99 |
| 1. Using your prosthesis to gently pat a dog or cat | 0 | 1 | 2 | 3 | 4 | 97 | 98 | 99 |
| 1. Using your prosthesis to deliver a soft or a firm touch when patting someone on the back | 0 | 1 | 2 | 3 | 4 | 97 | 98 | 99 |
| 1. Using your prosthesis in your physical and intimate relationships | 0 | 1 | 2 | 3 | 4 | 97 | 98 | 99 |
| 1. Using your prosthesis to hold a child | 0 | 1 | 2 | 3 | 4 | 97 | 98 | 99 |
| 1. Using your prosthesis to pick up a small child | 0 | 1 | 2 | 3 | 4 | 97 | 98 | 99 |

**[ASK IF USER AND USED PROSTHESIS IN PAST 4 WEEKS]**

Thinking about your experience over the last 4 weeks, how much do you agree with each of the following statements? Use any number from 0 to 4, where 0 is Do Not Agree At All and 4 is Agree Very Much.

|  | Do not agree at all |  |  |  | Agree very much | DON’T KNOW/ NOT SURE/NOT APPLICABLE [DO NOT READ] | REFUSED [DO NOT READ] |
| --- | --- | --- | --- | --- | --- | --- | --- |
| 1. My prosthesis is a part of me | 0 | 1 | 2 | 3 | 4 | 98 | 99 |
| 1. I feel more complete when wearing my prosthesis | 0 | 1 | 2 | 3 | 4 | 98 | 99 |
| 1. My prosthesis feels like a hand | 0 | 1 | 2 | 3 | 4 | 98 | 99 |
| 1. I look forward to removing my prosthesis so I can be more comfortable | 0 | 1 | 2 | 3 | 4 | 98 | 99 |
| 1. My prosthesis is an extension of my body | 0 | 1 | 2 | 3 | 4 | 98 | 99 |
| 1. I use my prosthesis to express myself | 0 | 1 | 2 | 3 | 4 | 98 | 99 |
| 1. When I take off my prosthesis, I feel a sense of loss | 0 | 1 | 2 | 3 | 4 | 98 | 99 |
| 1. Using my prosthesis slows me down | 0 | 1 | 2 | 3 | 4 | 98 | 99 |
| 1. Using my prosthesis requires concentration | 0 | 1 | 2 | 3 | 4 | 98 | 99 |
| 1. Using my prosthesis requires visual focus | 0 | 1 | 2 | 3 | 4 | 98 | 99 |
| 1. Using my prosthesis is not natural | 0 | 1 | 2 | 3 | 4 | 98 | 99 |
| 1. Using my prosthesis is clumsy | 0 | 1 | 2 | 3 | 4 | 98 | 99 |

**[ASK IF USER]** Using any number from 0 to 4, where 0 is Not at All and 4 is Very Much, to what extent did you feel each of the following over the last 4 weeks when you are **not** wearing a prosthesis?

**[ASK IF NON_USER]** Using any number from 0 to 4, where 0 is Not at All and 4 is Very Much, to what extent did you feel each of the following over the last 4 weeks?

|  | Not at All |  |  |  | Very Much | DON’T KNOW/ NOT SURE/NOT APPLICABLE [DO NOT READ] | REFUSED [DO NOT READ] |
| --- | --- | --- | --- | --- | --- | --- | --- |
| 1. Confident [Please pick any number from 0 to 4 to indicate how you felt, where 0 equals Not at All and 4 equals Very Much] | 0 | 1 | 2 | 3 | 4 | 98 | 99 |
| 1. Happy | 0 | 1 | 2 | 3 | 4 | 98 | 99 |
| 1. Whole | 0 | 1 | 2 | 3 | 4 | 98 | 99 |
| 1. Relieved | 0 | 1 | 2 | 3 | 4 | 98 | 99 |
| 1. Relaxed | 0 | 1 | 2 | 3 | 4 | 98 | 99 |
| 1. Free | 0 | 1 | 2 | 3 | 4 | 98 | 99 |
| 1. Vulnerable | 0 | 1 | 2 | 3 | 4 | 98 | 99 |
| 1. Incomplete | 0 | 1 | 2 | 3 | 4 | 98 | 99 |
| 1. Different from others | 0 | 1 | 2 | 3 | 4 | 98 | 99 |
| 1. Shy in public | 0 | 1 | 2 | 3 | 4 | 98 | 99 |
