# Supplementary material for: Structural validity and reliability of the patient experience measure: A new approach to assessing psychosocial experience of upper limb prosthesis users
Source: PLoS One. 2021 Dec 28;16(12):e0261865. doi: 10.1371/journal.pone.0261865 (PMC8714100; doi:10.1371/journal.pone.0261865)
Supplement: S3 Appendix — (DOCX) [file pone.0261865.s003.docx]

**S3 Appendix**

**Final Revised Patient Experience Measure**

**Social Interaction**

1. For this next question, please answer using any number from 0 to 2, where 0 is Not at All Confident and 2 is Very Confident. How confident were you in your ability to do each of the following using your prosthesis over the last 4 weeks?

|  | Not at All Confident |  | Very Confident | WOULD NOT DO/DO NOT DO | DON’T KNOW/ NOT SURE/NOT APPLICABLE |
| --- | --- | --- | --- | --- | --- |
| Using your prosthesis to grasp someone else's hand while walking without hurting them | 0 | 1 | 2 | 97 | 98 |
| Opening your terminal device when shaking hands | 0 | 1 | 2 | 97 | 98 |

1. Using any number from 0 to 2, where 0 is Not at All Comfortable and 2 is Very Comfortable, how comfortable were you with each of the following over the last 4 weeks?

|  | Not at All Comfortable |  | Very Comfortable | WOULD NOT DO/DO NOT DO | DON’T KNOW/ NOT SURE/NOT APPLICABLE |
| --- | --- | --- | --- | --- | --- |
| Using your prosthesis to grasp someone else's hand while walking without hurting them | 0 | 1 | 2 | 97 | 98 |
| Grasping with your prosthesis to shake hands with someone close to you | 0 | 1 | 2 | 97 | 98 |
| Grasping with your prosthesis to shake hands with someone you just met | 0 | 1 | 2 | 97 | 98 |
| Grasping with your prosthesis to shake hands with someone you know well | 0 | 1 | 2 | 97 | 98 |
| Using your prosthesis to gently squeeze someone else's hand | 0 | 1 | 2 | 97 | 98 |
| Using your prosthesis when embracing someone you care about | 0 | 1 | 2 | 97 | 98 |
| Using your prosthesis to convey a friendly or caring touch | 0 | 1 | 2 | 97 | 98 |
| Using your prosthesis to gently pat a dog or cat | 0 | 1 | 2 | 97 | 98 |
| Using your prosthesis to deliver a soft or a firm touch when patting someone on the back | 0 | 1 | 2 | 97 | 98 |
| Using your prosthesis in your physical and intimate relationships | 0 | 1 | 2 | 97 | 98 |
| Using your prosthesis to hold a child | 0 | 1 | 2 | 97 | 98 |
| Using your prosthesis to pick up a small child | 0 | 1 | 2 | 97 | 98 |

**Self-efficacy**

1. For this next question, please answer using any number from 0 to 2, where 0 is Not at All Confident and 2 is Very Confident. How confident were you in your ability to do each of the following using your prosthesis over the last 4 weeks?

|  | Not at All Confident |  | Very Confident | WOULD NOT DO/DO NOT DO | DON’T KNOW/ NOT SURE/NOT APPLICABLE |
| --- | --- | --- | --- | --- | --- |
| Using your prosthesis to carry a small object, such as a coin, without dropping it | 0 | 1 | 2 | 97 | 98 |
| Using your prosthesis to pick up an open plastic water bottle without dropping or crushing it | 0 | 1 | 2 | 97 | 98 |
| Using your prosthesis to drink from a paper cup without dropping or crushing it | 0 | 1 | 2 | 97 | 98 |
| Using your prosthesis to pick up a Ritz cracker without breaking it | 0 | 1 | 2 | 97 | 98 |
| Using your prosthesis to eat with a knife and fork while in a restaurant | 0 | 1 | 2 | 97 | 98 |
| Holding a dinner glass using your prosthesis | 0 | 1 | 2 | 97 | 98 |
| Tying a knot using your prosthesis | 0 | 1 | 2 | 97 | 98 |
| Using your prosthesis to carry a slippery object, such as a silk scarf or tie, without dropping it | 0 | 1 | 2 | 97 | 98 |
| Using your prosthesis to pick up fragile objects | 0 | 1 | 2 | 97 | 98 |
| Using your prosthesis to carry a laundry basket | 0 | 1 | 2 | 97 | 98 |
| Trying new tasks with your prosthesis | 0 | 1 | 2 | 97 | 98 |

1. Using any number from 0 to 2, where 0 is Not at All Comfortable and 2 is Very Comfortable, how comfortable were you with each of the following over the last 4 weeks?

|  | Not at All Comfortable |  | Very Comfortable | WOULD NOT DO/DO NOT DO | DON’T KNOW/ NOT SURE/NOT APPLICABLE |
| --- | --- | --- | --- | --- | --- |
| Using your prosthesis to hold a child | 0 | 1 | 2 | 97 | 98 |

**Embodiment**

1. Thinking about your experience over the last 4 weeks, how much do you agree with each of the following statements? Use any number from 0 to 2, where 0 is Do Not Agree At All and 2 is Agree Very Much.

|  | Do not Agree |  | Agree Very Much | DON’T KNOW/ NOT SURE/NOT APPLICABLE |
| --- | --- | --- | --- | --- |
| My prosthesis is a part of me | 0 | 1 | 2 | 98 |
| I feel more complete when wearing my prosthesis | 0 | 1 | 2 | 98 |
| My prosthesis feels like a hand | 0 | 1 | 2 | 98 |
| My prosthesis is an extension of my body | 0 | 1 | 2 | 98 |
| I use my prosthesis to express myself | 0 | 1 | 2 | 98 |

**Intuitiveness**

1. Thinking about your experience over the last 4 weeks, how much do you agree with each of the following statements? Use any number from 0 to 3, where 0 is Agree Very Much and 3 is Do Not Agree At All.

|  | Do not agree  at all | |  | Agree very much | DON’T KNOW/ NOT SURE/NOT APPLICABLE |
| --- | --- | --- | --- | --- | --- |
| Using my prosthesis slows me down | 3 | 2 | 1 | 0 | 98 |
| Using my prosthesis requires concentration | 3 | 2 | 1 | 0 | 98 |
| Using my prosthesis is not natural | 3 | 2 | 1 | 0 | 98 |
| Using my prosthesis is clumsy | 3 | 2 | 1 | 0 | 98 |

*these items have already been reverse coded

**Wellbeing**

1. Using any number from 0 to 3, where 0 is Not at All and 3 is Very Much, to what extent did you feel each of the following over the last 4 weeks when you are **not** wearing a prosthesis?

|  | Not at All |  |  | Very Much | DON’T KNOW/ NOT SURE/NOT APPLICABLE |
| --- | --- | --- | --- | --- | --- |
| Confident | 0 | 1 | 2 | 3 | 98 |
| Happy | 0 | 1 | 2 | 3 | 98 |
| Whole | 0 | 1 | 2 | 3 | 98 |
| Relieved | 0 | 1 | 2 | 3 | 98 |
| Relaxed | 0 | 1 | 2 | 3 | 98 |
| Free | 0 | 1 | 2 | 3 | 98 |

**Self-consciousness**

1. Using any number from 0 to 2, where 0 is Very Much and 2 is Not At All, to what extent did you feel each of the following over the last 4 weeks when you are **not** wearing a prosthesis?

|  | Not at  all |  | Very  Much | DON’T KNOW/ NOT SURE/NOT APPLICABLE |
| --- | --- | --- | --- | --- |
| Vulnerable | 2 | 1 | 0 | 98 |
| Incomplete | 2 | 1 | 0 | 98 |
| Different from others | 2 | 1 | 0 | 98 |
| Shy in public | 2 | 1 | 0 | 98 |

*these items have already been reverse coded
