# Supplementary material for: Structural validity and reliability of the patient experience measure: A new approach to assessing psychosocial experience of upper limb prosthesis users
Source: PLoS One. 2021 Dec 28;16(12):e0261865. doi: 10.1371/journal.pone.0261865 (PMC8714100; doi:10.1371/journal.pone.0261865)
Supplement: S4 Appendix — (DOCX) [file pone.0261865.s004.docx]

**S4 Appendix. Scoring Tables**

|  | Those 65 or under | | Those over 65 | |
| --- | --- | --- | --- | --- |
| Raw summed score | T score | Standard Error | T score | Standard Error |
| 0 | 31.58 | 7.16 | 31.93 | 7.20 |
| 1 | 36.59 | 4.12 | 36.97 | 4.12 |
| 2 | 39.79 | 3.08 | 40.21 | 3.08 |
| 3 | 41.86 | 2.62 | 42.29 | 2.62 |
| 4 | 43.48 | 2.39 | 43.91 | 2.39 |
| 5 | 44.87 | 2.23 | 45.29 | 2.23 |
| 6 | 46.10 | 2.12 | 46.52 | 2.12 |
| 7 | 47.26 | 2.08 | 47.64 | 2.04 |
| 8 | 48.33 | 2.00 | 48.68 | 2.00 |
| 9 | 49.37 | 2.00 | 49.72 | 1.96 |
| 10 | 50.37 | 1.96 | 50.68 | 1.93 |
| 11 | 51.38 | 1.96 | 51.68 | 1.93 |
| 12 | 52.38 | 1.96 | 52.65 | 1.93 |
| 13 | 53.42 | 1.96 | 53.65 | 1.96 |
| 14 | 54.42 | 2.00 | 54.65 | 2.00 |
| 15 | 55.5 | 2.04 | 55.69 | 2.04 |
| 16 | 56.61 | 2.12 | 56.80 | 2.08 |
| 17 | 57.81 | 2.19 | 57.96 | 2.19 |
| 18 | 59.15 | 2.35 | 59.31 | 2.35 |
| 19 | 60.69 | 2.58 | 60.85 | 2.58 |
| 20 | 62.70 | 3.00 | 62.85 | 3.00 |
| 21 | 65.78 | 4.04 | 65.89 | 4.04 |
| 22 | 70.70 | 7.12 | 70.82 | 7.12 |

**Table 1. Social Interaction scale scoring, split by DIF age groups.**

|  | Male, Unilateral | | Male, Bilateral | | Female, Unilateral | | Female, Bilateral | |
| --- | --- | --- | --- | --- | --- | --- | --- | --- |
| Raw summed score | T score | Standard Error | T score | Standard Error | T score | Standard Error | T score | Standard Error |
| 0 | 21.76 | 9.87 | 21.76 | 9.87 | 21.55 | 9.87 | 21.55 | 9.87 |
| 1 | 28.85 | 5.77 | 28.80 | 5.72 | 28.59 | 5.72 | 28.53 | 5.72 |
| 2 | 33.47 | 4.36 | 33.42 | 4.30 | 33.15 | 4.30 | 33.10 | 4.30 |
| 3 | 36.57 | 3.73 | 36.46 | 3.73 | 36.20 | 3.67 | 36.09 | 3.67 |
| 4 | 38.93 | 3.36 | 38.82 | 3.36 | 38.51 | 3.31 | 38.40 | 3.31 |
| 5 | 40.92 | 3.10 | 40.76 | 3.10 | 40.45 | 3.10 | 40.34 | 3.04 |
| 6 | 42.65 | 2.94 | 42.50 | 2.94 | 42.18 | 2.94 | 42.02 | 2.89 |
| 7 | 44.23 | 2.83 | 44.02 | 2.78 | 43.76 | 2.83 | 43.60 | 2.78 |
| 8 | 45.70 | 2.73 | 45.49 | 2.73 | 45.23 | 2.73 | 45.02 | 2.68 |
| 9 | 47.12 | 2.68 | 46.85 | 2.62 | 46.64 | 2.68 | 46.38 | 2.62 |
| 10 | 48.43 | 2.62 | 48.17 | 2.62 | 47.96 | 2.62 | 47.69 | 2.62 |
| 11 | 49.74 | 2.62 | 49.43 | 2.57 | 49.27 | 2.62 | 48.95 | 2.57 |
| 12 | 51.00 | 2.57 | 50.69 | 2.57 | 50.58 | 2.62 | 50.27 | 2.57 |
| 13 | 52.31 | 2.57 | 51.95 | 2.57 | 51.89 | 2.62 | 51.53 | 2.62 |
| 14 | 53.57 | 2.62 | 53.26 | 2.62 | 53.21 | 2.62 | 52.84 | 2.62 |
| 15 | 54.89 | 2.62 | 54.57 | 2.68 | 54.52 | 2.68 | 54.20 | 2.68 |
| 16 | 56.25 | 2.68 | 55.94 | 2.73 | 55.88 | 2.73 | 55.57 | 2.73 |
| 17 | 57.67 | 2.78 | 57.41 | 2.78 | 57.35 | 2.78 | 57.04 | 2.83 |
| 18 | 59.14 | 2.89 | 58.93 | 2.94 | 58.88 | 2.89 | 58.61 | 2.94 |
| 19 | 60.82 | 2.99 | 60.66 | 3.10 | 60.56 | 3.04 | 60.35 | 3.10 |
| 20 | 62.66 | 3.20 | 62.55 | 3.31 | 62.39 | 3.25 | 62.34 | 3.31 |
| 21 | 64.81 | 3.57 | 64.86 | 3.62 | 64.60 | 3.57 | 64.60 | 3.67 |
| 22 | 67.59 | 4.15 | 67.75 | 4.20 | 67.43 | 4.15 | 67.54 | 4.25 |
| 23 | 71.90 | 5.56 | 72.16 | 5.62 | 71.74 | 5.56 | 72.00 | 5.62 |
| 24 | 78.67 | 9.76 | 78.98 | 9.76 | 78.51 | 9.76 | 78.88 | 9.82 |

**Table 2. Self-efficacy scale scoring, split by DIF gender and laterality groups.**

|  | Those 65 or under | | Those over 65 | |
| --- | --- | --- | --- | --- |
| Raw summed score | T score | Standard Error | T score | Standard Error |
| 0 | 26.28 | 7.15 | 26.46 | 7.18 |
| 1 | 31.75 | 4.43 | 32.01 | 4.50 |
| 2 | 36.03 | 3.72 | 36.55 | 3.91 |
| 3 | 39.56 | 3.57 | 40.57 | 3.80 |
| 4 | 42.88 | 3.42 | 44.14 | 3.46 |
| 5 | 45.89 | 3.27 | 47.08 | 3.20 |
| 6 | 48.72 | 3.20 | 49.76 | 3.16 |
| 7 | 51.58 | 3.35 | 52.59 | 3.39 |
| 8 | 54.90 | 3.72 | 56.01 | 3.80 |
| 9 | 59.40 | 4.61 | 60.55 | 4.54 |
| 10 | 65.24 | 7.29 | 66.25 | 7.22 |

**Table 3. Embodiment scale scoring, no DIF groups.**

|  | Those 65 or under | |
| --- | --- | --- |
| Raw summed score | T score | Standard Error |
| 0 | 25.87 | 10.46 |
| 1 | 32.90 | 5.83 |
| 2 | 37.24 | 4.34 |
| 3 | 40.10 | 3.77 |
| 4 | 42.44 | 3.54 |
| 5 | 44.61 | 3.49 |
| 6 | 46.73 | 3.54 |
| 7 | 48.95 | 3.66 |
| 8 | 51.47 | 3.94 |
| 9 | 54.38 | 4.29 |
| 10 | 58.10 | 4.91 |
| 11 | 63.41 | 6.34 |
| 12 | 71.19 | 10.80 |

**Table 4. Intuitiveness scale scoring, no DIF groups.**

|  | Prosthesis Nonusers | | Prosthesis Users | | |
| --- | --- | --- | --- | --- | --- |
| Raw summed score | T score | Standard Error | T score | Standard Error |  |
| 0 | 20.73 | 9.66 | 20.89 | 9.72 |  |
| 1 | 27.48 | 5.56 | 27.64 | 5.56 |  |
| 2 | 29.25 | 4.16 | 32.06 | 4.21 |  |
| 3 | 34.65 | 3.59 | 34.97 | 3.64 |  |
| 4 | 36.89 | 3.22 | 37.20 | 3.27 |  |
| 5 | 38.71 | 2.96 | 39.12 | 3.07 |  |
| 6 | 40.32 | 2.81 | 40.79 | 2.86 |  |
| 7 | 41.72 | 2.65 | 42.34 | 2.75 |  |
| 8 | 43.07 | 2.60 | 43.75 | 2.65 |  |
| 9 | 44.32 | 2.55 | 45.05 | 2.60 |  |
| 10 | 45.57 | 2.55 | 46.40 | 2.60 |  |
| 11 | 46.81 | 2.65 | 47.70 | 2.65 |  |
| 12 | 48.11 | 2.65 | 49.05 | 2.70 |  |
| 13 | 49.51 | 2.75 | 50.50 | 2.81 |  |
| 14 | 51.07 | 2.96 | 52.16 | 3.01 |  |
| 15 | 52.94 | 3.33 | 54.09 | 3.33 |  |
| 16 | 55.44 | 3.90 | 56.63 | 3.95 |  |
| 17 | 59.39 | 5.35 | 60.58 | 5.40 |  |
| 18 | 65.88 | 9.56 | 67.13 | 9.56 |  |

**Table 5. Wellbeing scale scoring, split by DIF prosthesis use groups.**

| Raw summed score | T score | Standard Error |
| --- | --- | --- |
| 0 | 29.07 | 8.58 |
| 1 | 35.44 | 5.19 |
| 2 | 40.22 | 4.29 |
| 3 | 44.06 | 4.11 |
| 4 | 47.72 | 4.02 |
| 5 | 51.33 | 4.06 |
| 6 | 55.12 | 4.29 |
| 7 | 59.86 | 5.24 |
| 8 | 66.32 | 8.62 |

**Table 6. Self-consciousness scale scoring, no DIF groups.**
