# Supplementary material for: Structural validity and reliability of the patient experience measure: A new approach to assessing psychosocial experience of upper limb prosthesis users
Source: PLoS One. 2021 Dec 28;16(12):e0261865. doi: 10.1371/journal.pone.0261865 (PMC8714100; doi:10.1371/journal.pone.0261865)
Supplement: Attachment — Submitted filename: Response to Reviewers.docx [file pone.0261865.s006.docx]

**Journal Requirements:

Please review your reference list to ensure that it is complete and correct. If you have cited papers that have been retracted, please include the rationale for doing so in the manuscript text, or remove these references and replace them with relevant current references. Any changes to the reference list should be mentioned in the rebuttal letter that accompanies your revised manuscript. If you need to cite a retracted article, indicate the article’s retracted status in the References list and also include a citation and full reference for the retraction notice.**

We have reviewed the reference list and are not aware of any references that have been retracted.

**Review Comments to the Author

Reviewer #1: Those comments have been addressed where appropriate, either in the analysis above or in an individual reply to the exporter concerned.**

Thank you – we are glad the revisions have addressed all concerns.

**Reviewer #2: Thank you for revising the previous comments well. The manuscript looks good, though it still needs some minor revision. abstract well written, Introduction corrections are acceptable.**

Thank you, we appreciate the Reviewer’s feedback.

**In the method section, small changes are needed, which are as follows:
What do you mean by table 1 where the type of prosthesis is mentioned?**

Table 1 reports the ’primary prosthesis type’ that is used for the pilot and cognitive samples. We elected to name the category this way because some participants have multiple types of prostheses. The primary type is the type that is used most often. We believe the different types listed (Body-powered, myoelectric, hybrid, etc) will be familiar categories and that this is useful descriptive information about the sample of persons with amputation included in this study. We added a definition of “primary” to the table legend.

**If possible, state the length of time since the amputation. The duration of prosthesis use can also be beneficial.**
Table 2 provides time since amputation. We did not collect this data for participants in the pilot and cognitive sample**.** We do not have data on the number of years of prosthesis use.

**If possible, summarize the factor analysis and rasch analysis. Written too long.**

We agree that the results of the factor and Rasch analyses are long. We deleted some text and referred to information available in tables, but are reluctant to remove more key information that explains our approach.

**The number of titles in the method section is high. You can, for example, use a title for the two sections Floor and ceiling and Transformation and scoring.**

Thank-you for this suggestion. We have combined subsection titles as recommended. We also removed EFA results and CFA results and left these under the overall section ‘Factor Analysis’.

**In Table 2, when the Military option is zero for everyone, it is better to delete this row altogether.**

We have deleted this row at the reviewer’s request.

**I do not understand anything from Table 2 in general? For example, for Body-powered, the number 155 is written with a standard deviation of 73, I do not understand exactly what it means. please clarify**.

Table 2 presents continuous variables as mn(sd) and categorical variables as N(%). These headings are provided within the table. For the example provided, the number of body-powered users (a categorical variable) was 155, which was 73.5% of the sample.
